# Influence of Tempeh, Daidzein, Probiotics, and Their Combination on Magnesium Status and Hematological Ratios in a Postmenopausal Osteoporotic Animal Model

**DOI:** 10.3390/nu17182917

**Published:** 2025-09-09

**Authors:** Iskandar Azmy Harahap, Omar Salem, Rifaldi Lutfi Fahmi, Naglaa Ahmed, Natalia Leciejewska, Joanna Suliburska

**Affiliations:** 1 Department of Human Nutrition and Dietetics, Faculty of Food Science and Nutrition, Poznan University of Life Sciences, 60-624 Poznan, Poland; 2 Postgraduate School, Faculty of Food Science and Nutrition, Poznan University of Life Sciences, 60-624 Poznan, Poland; 3 Department of Animal Physiology, Biochemistry and Biostructure, Faculty of Veterinary Medicine and Animal Science, Poznan University of Life Sciences, 60-637 Poznan, Poland

**Keywords:** phytoestrogens, bioactive compounds, magnesium homeostasis, inflammatory indices, postmenopausal osteoporosis

## Abstract

Background/Objectives: Postmenopausal osteoporosis disrupts magnesium homeostasis and hematological balance, contributing to systemic inflammation and metabolic alterations. This study aimed to assess the effects of dietary interventions—tempeh, daidzein, probiotics, and their combinations—on magnesium status and hematological ratios in a postmenopausal osteoporotic Wistar rat model. Methods: Sixty-four rats were divided into one sham group (*n* = 8) and seven ovariectomized (OVX) groups (*n* = 56), with different modified diets administered for six weeks. In addition, one of the groups received alendronate bisphosphonate as a pharmacological reference to benchmark the dietary interventions against standard anti-osteoporotic therapy. Magnesium levels in the tissues and feces, along with blood hematological ratios (neutrophil-to-lymphocyte ratio (NLR), monocyte-to-lymphocyte ratio (MLR), platelet-to-lymphocyte ratio (PLR), and triglyceride-to-glucose index (TyG)), were evaluated. Results: The results revealed that a combination of tempeh and probiotics (OTL) significantly increased magnesium levels in the feces, spleen, and hair, while reducing liver magnesium levels. Compared to the standard groups (S and O), the hematological analysis revealed that the daidzein group (OD) had the highest MLR, while the OTL group exhibited the lowest TyG index. The alendronate bisphosphonate (OB) intervention showed no significant effect on tissue magnesium levels, feces magnesium levels, or hematological ratios. Correlation analysis revealed a strong negative association between spleen magnesium levels and the PLR (*r* = −0.626) and a positive relationship between liver magnesium levels and TyG (*r* = 0.422). Conclusions: The authors of this study concludes that while ovariectomy significantly altered magnesium status and hematological ratios, the dietary combination of tempeh, daidzein, and probiotics did not demonstrate an apparent beneficial effect on magnesium status or inflammatory ratios in a postmenopausal osteoporotic rat model. However, the findings highlight interesting aspects of magnesium status and its correlations with metabolic and inflammatory parameters, warranting further investigation.

## 1. Introduction

The global demographic shift toward an ageing population presents significant public health challenges, with the proportion of individuals aged 60 and older expected to double to over 2 billion by 2050, predominantly in low- and middle-income countries [1]. This trend is accompanied by a rise in age-related conditions, particularly osteoporosis and metabolic disorders, which exacerbate healthcare burdens. Osteoporosis, marked by reduced bone mass and structural deterioration, increases fracture risk, disability, and healthcare costs while often co-occurring with metabolic disorders like insulin resistance and dyslipidemia [2,3,4]. Among the aging population, women are especially vulnerable to such problems due to the hormonal changes of menopause, leading to accelerated bone loss, heightened osteoporosis risk, and systemic inflammation, which disrupts glucose and lipid metabolism [5].

Postmenopausal osteoporosis is a critical health issue that disproportionately affects women after menopause, primarily driven by a sharp decline in estrogen levels. Estrogen plays a central role in regulating bone remodeling by maintaining a balance between bone resorption and formation [6]. Its deficiency triggers an increase in osteoclast activity and a concomitant decrease in osteoblast function, resulting in accelerated bone loss and compromised bone microarchitecture [7,8]. This imbalance leads to reduced bone mineral density (BMD), disrupted calcium and magnesium homeostasis, and a heightened risk of fractures, particularly in the hip, spine, and wrist. Calcium and magnesium, which are essential minerals for bone strength and metabolic stability, are often dysregulated in postmenopausal osteoporosis, further aggravating skeletal fragility [9,10].

Beyond its direct impact on bone health, postmenopausal osteoporosis has systemic consequences that extend to hematological balance, inflammation, and metabolism. Osteoporosis is closely linked to alterations in hematological indices such as the neutrophil-to-lymphocyte ratio (NLR), platelet-to-lymphocyte ratio (PLR), and triglyceride-to-glucose index (TyG), which serve as markers of systemic inflammation and metabolic health [11,12]. Elevated NLR and PLR values reflect a pro-inflammatory state [11], while an increased TyG index suggests insulin resistance and dysregulated lipid metabolism [12], both of which exacerbate the progression of osteoporosis and other metabolic disorders [13]. Recent evidence further supports the TyG index as a significant predictor of postmenopausal osteoporosis, emphasizing its value in integrating metabolic health assessment into osteoporosis management strategies [14]. The interplay between disrupted hematological balance, chronic inflammation, and altered metabolic pathways forms a vicious cycle that accelerates bone loss and systemic deterioration [15]. These phenomena emphasize the importance of addressing skeletal and extra-skeletal factors to develop comprehensive therapeutic strategies for managing postmenopausal osteoporosis and its systemic impact.

Magnesium is a vital mineral essential for bone health, immune regulation, and metabolic processes, with approximately 60% of the body’s magnesium stored in the bone to support structural integrity and remodeling activities [16]. In postmenopausal osteoporosis, estrogen deficiency disrupts magnesium homeostasis by reducing its absorption and increasing renal excretion, thereby exacerbating bone loss, systemic inflammation, and metabolic dysfunction [17,18]. Magnesium deficiency has been linked to elevated pro-inflammatory cytokine levels, oxidative stress, and insulin resistance, further compounding the risks of osteoporosis and associated metabolic disorders [19]. These disruptions underscore the importance of magnesium in mitigating postmenopausal health challenges, highlighting its potential role as a therapeutic target for improving bone density and reducing systemic inflammation.

Phytoestrogens, particularly isoflavones like daidzein, which is derived from soy products, are plant-based compounds that structurally resemble estrogen and can exert estrogenic or anti-estrogenic effects depending on hormonal status, making them promising alternatives for addressing postmenopausal osteoporosis [20,21]. Tempeh, a fermented soy product, is a rich source of the isoflavones and bioactive peptides produced during fermentation, which not only mimic estrogenic activity but also have the potential to improve magnesium metabolism and reduce systemic inflammation, critical factors in mitigating osteoporosis-related risks [22,23]. Furthermore, probiotics, particularly strains like *Lactobacillus acidophilus*, play a pivotal role in enhancing mineral bioavailability by modulating gut microbiota, improving intestinal absorption, and reducing inflammatory markers, collectively supporting bone health and immune regulation [24,25].

While previous research has examined the individual effects of magnesium metabolism, isoflavones, and probiotics on health, particularly in healthy subjects [26], significant gaps remain in understanding their combined impact on postmenopausal health, especially within an osteoporotic model. A prior study in healthy older female rats demonstrated that tempeh consumption influenced magnesium concentrations in various tissues, albeit without significant effects from pure daidzein, genistein, or probiotics on magnesium status [26]. Building on these findings, the current study uniquely explores the combined impact of tempeh, daidzein, genistein, and probiotics on magnesium metabolism and various hematological parameters in a postmenopausal osteoporotic model. By examining this specific combination, this study provides novel insights into how these dietary components interact to enhance magnesium absorption, mitigate inflammation, and regulate metabolic pathways.

This study aimed to evaluate the effects of dietary interventions involving tempeh, daidzein, probiotics, and their combination on magnesium status and hematological ratios in a postmenopausal osteoporotic animal model. Recognizing magnesium’s critical role in bone health, immune regulation, and systemic metabolism, the authors hypothesized that incorporating phytoestrogen sources and probiotics into the diet would improve magnesium status, reduce systemic inflammation, and restore metabolic balance in osteoporosis.

## 2. Materials and Methods

### 2.1. Materials

Three-month-old female Wistar rats were purchased from the Instytut Biologii Doświadczalnej im. M. Nenckiego PAN (Warsaw, Poland). The animals were provided with a standard diet based on an AIN-93M formulation, obtained from Zoolab (Sędziszów, Poland). Key dietary ingredients included Augusta variety soybeans, supplied by the Uniwersytet Przyrodniczy w Poznaniu (Poznan, Poland), and purified daidzein, obtained from Gentaur Molecular Products BVBA (Kampenhout, Belgium). Calcium citrate tetrahydrate, an essential component of this dietary intervention, was purchased from Warchem Sp. z o.o. (Warsaw, Poland). Tempeh flour for diet intervention was also prepared using the Rhizopus oligosporus NRRL 2710 strain (Agricultural Research Service Culture Collection, Peoria, IL, USA). The probiotic strain *Lactobacillus acidophilus* DSM20079 for diet intervention was obtained from the Leibniz Institute DSMZ (Deutsche Sammlung von Mikroorganismen und Zellkulturen GmbH, Braunschweig, Germany), following the protocol outlined in a prior study [27,28,29].

### 2.2. Ethical Considerations in Animal Research

The Lokalna Komisja Etyczna (Poznan, Poland) granted ethical approval for this study under registration number 21/2021. The research was conducted in strict adherence to established guidelines and regulations, including the National Institutes of Health’s Handbook for the Care and Use of Laboratory Animals (NIH Publication No. 80-23, Revised 1978), Directive 2010/63/EU of the European Parliament and Council (22 September 2010) on the protection of animals used in scientific research, and relevant Polish laws. Additionally, all animal procedures were carried out in full compliance with the standards set by the Poznań University of Life Sciences, Poland, and followed the principles outlined in the Animal Research: Reporting of In Vivo Experiments (ARRIVE) guidelines.

### 2.3. Preparing the Animal Laboratory Environment

**Figure 1:**
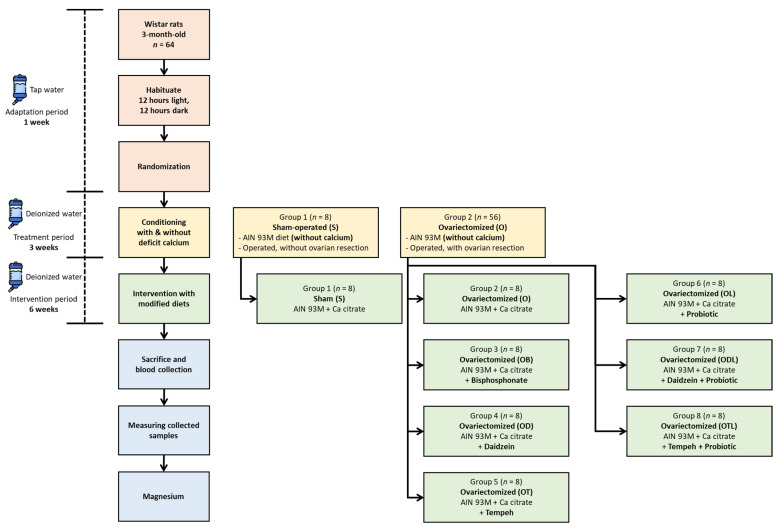
Flowchart showing the design of the experiment.

The female Wistar rats were housed under secure and controlled conditions in the Animal Laboratory at Uniwersytet Przyrodniczy w Poznaniu (Poznan, Poland). The choice of 3-month-old animals was based on their suitability as an established model for inducing estrogen deficiency-related bone loss through ovariectomy. At this early adult stage, ovariectomy reliably triggers metabolic and skeletal changes that are consistent with postmenopausal osteoporosis within a relatively short timeframe, enabling efficient and reproducible intervention studies [30]. Figure 1 presents a flowchart depicting the research process.

Wistar rats, in particular, were selected due to their proven sensitivity to estrogen and their consistent sexual cycle stability, which ensures experimental robustness and reliability in osteoporosis research [31]. The rats were kept in a chamber with a stable temperature of 21 ± 2 °C, a relative humidity of 55–65%, and a 12-h light/dark cycle. They were housed in pairs in stainless steel cages with an enamel coating to minimize electromagnetic interference during the adaptation and intervention phases.

The rats were given one week to acclimatize to the laboratory environment before the trial began. During this period and throughout the experiment, Labofeed B (Żurawia, Kcynia, Poland) and fresh water were freely provided to the rats. Labofeed B complies with the nutritional requirements for adult rats, as outlined by the National Research Council of Poland.

To minimize stress, the number of people interacting with the rats and the duration of these interactions were kept to a minimum during the study. Additionally, the rats had continuous access to veterinary care throughout the experiment.

### 2.4. Bilateral Ovariectomy Procedure

Upon completion of the adaptation period, the rats were separated into two distinct groups: a sham operation group (*n* = 8) and a bilateral ovariectomy group (*n* = 56). A highly skilled physician with expertise in animal ovariectomy performed all the surgeries. During the operations, anesthesia was induced using a mixture of ketamine and Cepetor. Meticulous attention was paid to maintaining strict cleanliness and sterile conditions throughout the procedures. To facilitate the surgeries, the dorsal region of each rat was shaved, and a linear incision of approximately 10–15 mm was made at the designated surgical site.

After the surgical procedure, each rat was placed on a heated mat, maintained at approximately 30 °C, to create a comfortable and supportive environment for recovery. Continuous monitoring was conducted for seven days post-surgery to ensure the well-being of all the rats. Following the surgeries, the rats were provided with a semi-synthetic diet, formulated according to the AIN-93M guidelines [32]. Additionally, each rat had free access to tap water during this period.

### 2.5. Assigning Rats to Experimental Groups

After a week-long recovery period, the initial body weights of the rats were measured using a calibrated scale. This step is essential for adequately randomizing the experimental groups, ensuring that each group starts the study from a similar baseline. The rats that underwent ovariectomy were randomly assigned to two groups, each consisting of eight animals, based on their body weight. Randomizing animals in an experimental design is crucial as it reduces the potential for bias and ensures that the groups are comparable at the start of the study [33]. Previous research has shown that having at least eight rats per group provides sufficient statistical power to detect the significant effects of the intervention [34].

### 2.6. Establishing Calcium Deficiency

To ensure comparable baseline metabolic conditions, the rats in the sham group (S, *n* = 8) were initially fed a calcium-deficient standard diet for three weeks, alongside the ovariectomized rats (Group 2, *n* = 56). This short-term calcium restriction was applied to standardize mineral depletion across all animals prior to the experimental interventions, as previous studies have shown that a duration of three weeks is sufficient to induce calcium deficiency [35]. Following this period, animals were allocated into eight groups: the sham group resumed a normal calcium diet, while the remaining ovariectomized groups continued under their respective experimental diets. Throughout the three-week baseline period, daily food intake was monitored, and unlimited access to deionized water was provided. This strategy allowed the study to establish a consistent baseline mineral status, minimizing confounding effects on magnesium metabolism and hematological parameters.

### 2.7. Dietary Intervention Strategy

Following the induction of calcium deficiency, the sham (S) and ovariectomized (O) groups were provided with a standard diet along with calcium citrate tetrahydrate. The ovariectomized group was further divided into seven subgroups. The first subgroup received a standard diet along with calcium citrate (O). The second subgroup (OB) was given a standard diet along with calcium citrate and alendronate bisphosphonate (3 mg/kg of body weight, weekly adjusted). Alendronate bisphosphonate was included as a pharmacological reference, since it is a well-established anti-resorptive agent for postmenopausal osteoporosis. Its use in ovariectomized rat models has consistently demonstrated benefits on bone remodeling and fracture healing [36]. The third subgroup (OD) received a standard diet along with calcium citrate and daidzein (100 mg/kg of standard diet). The fourth subgroup (OT) was provided with a standard diet containing calcium citrate and tempeh flour (250 g/kg of standard diet). The fifth subgroup (OL) was given an AIN-93M diet along with *L*. *acidophilus* DSM079 at a dosage of 10^10^ CFU/day. The sixth subgroup (ODL) was given an AIN-93M diet along with daidzein and *L. acidophilus* DSM079. The seventh subgroup (OTL) received an AIN-93M diet along with tempeh and *L. acidophilus* DSM079. During the six-week intervention period, the rats’ food intake was monitored daily, and each rat was consistently provided with unlimited access to deionized water.

The design of the intervention period and dosage was based on both scientific rationale and translational considerations. A six-week feeding trial was selected, a duration that is frequently applied in rodent models to examine the influence of dietary isoflavones and probiotics on skeletal and metabolic outcomes [37,38]. Although longer experiments could provide additional information on long-term adaptations, this timeframe has been demonstrated to be sufficient for detecting measurable changes in postmenopausal animal models.

In this study, the doses of daidzein and tempeh were selected based on their isoflavone composition, as determined through laboratory analysis. Quantification of the tempeh preparation revealed that 250 g of tempeh contained approximately 10 mg of daidzein equivalents. Accordingly, the dietary formulation incorporated 250 g/kg of tempeh flour, which corresponded to ~10 mg/kg of daidzein. To ensure comparability between interventions, the pure daidzein treatment was adjusted to provide an equivalent concentration (10 mg/kg) when incorporated into the AIN-93M diet. This approach allowed for the incorporation of precise amounts of tempeh flour or daidzein into the diets and ensured that the diets remained nutritionally comparable. The isoflavones in tempeh are predominantly present as glycoside conjugates, which are hydrolyzed by the intestinal microbiota to release the bioactive aglycone forms, including daidzein, during digestion [39]. In contrast, the pure daidzein used in this study was supplied in its aglycone form, ensuring a defined dose of the active compound. The distinction between the conjugated isoflavones in tempeh and the aglycone daidzein in the pure supplement was carefully considered, as both the form and bioavailability of isoflavones can influence their biological effects. The chosen dosages were consistent with previously reported levels that were shown to exert beneficial effects on bone health in ovariectomized rodent models [40,41].

The probiotic dose was chosen in light of previous findings, where *L. acidophilus* at 10^9^ CFU/day effectively influenced osteoclastogenic activity in ovariectomized mice [42], while human trials showed a more pronounced effect at 10^10^ CFU/day compared with lower intakes [43]. Therefore, *L. acidophilus* was administered at 10^10^ CFU/day in the present study to align with the most effective level demonstrated in clinical research.

### 2.8. Tracking Dietary Intake

During the adaptation phase, the rats in each group were weighed weekly using a calibrated scale. Daily food intake was carefully recorded at the cage level by measuring the amount of food provided and subtracting the leftovers. The average intake per rat was calculated by dividing the total cage consumption by two. This approach, commonly applied in pair-housed rodent studies, allows for reliable estimation of individual dietary intake while avoiding the stress associated with single housing in metabolic cages [44]. Additionally, each rat was weighed weekly, which enabled the detection of deviations in growth or consumption, ensuring that all animals received adequate nutrition throughout the study. Fresh food and water were supplied daily, and leftovers were promptly removed to maintain consistent dietary conditions and prevent spoilage.

### 2.9. Euthanizing the Rats

At the end of the intervention phase, the rats underwent a fasting period of 4–6 h to minimize the impact of recent food intake on subsequent weight measurements. During this fasting period, their body weight was recorded. Following the weight assessment, the rats were euthanized by decapitation, a widely accepted and ethically sound method for experimental animals. Decapitation ensures a quick and painless death, thereby minimizing any potential distress. Blood samples were collected via cardiac puncture to analyze the hematological parameters. All animals completed the study; no deaths, exclusions, or losses occurred. All biochemical, hematological, and tissue magnesium measurements used the full per-group sample sizes (sham, *n* = 8; each OVX group, *n* = 8).

### 2.10. Measurement of Magnesium Levels in Diets, Tissues, and Feces

Magnesium concentrations in the diets and feces were determined by ashing the samples at 450 °C in a muffle furnace until fully mineralized. Specifically, 2 g of each diet and 1 g of feces were processed. The resulting ash was dissolved in 1N nitric acid (Suprapure, Merck, Kenilworth, NJ, USA), and magnesium levels were measured using flame atomic absorption spectrometry (AAS-3, Carl Zeiss, Jena, Germany).

For tissue analysis, including the spleen (μg/g), femur (mg/g), heart (μg/g), liver (μg/g), kidney (μg/g), and hair (μg/g), the samples were digested with 65% (*w*/*w*) ultrapure nitric acid (HNO_3_, Merck, Kenilworth, NJ, USA) in a microwave digestion system (Speedwave Xpert, Berghof, Eningen, Germany), following the protocol used by Harahap et al. [26]. Post-digestion, the solutions were diluted with deionized water, and magnesium concentrations were quantified using the same spectrometry technique (AAS-3, Carl Zeiss, Jena, Germany).

Magnesium levels in all samples were measured at a wavelength of 285.2 nm. To ensure the accuracy and reliability of the method, certified reference materials were utilized: soybean flour INCT-SBF-4 (Institute of Nuclear Chemistry and Technology, Warsaw, Poland) for diet analysis, achieving 98% accuracy, and bovine liver NIST-1577C (Sigma-Aldrich, Saint Louis, MO, USA) for tissue analysis, achieving 91% accuracy. To calculate the dietary magnesium intake, the concentration of magnesium in each experimental diet (mg/g) was multiplied by the mean weekly food consumption (g/week/rat) for each group. The resulting values were expressed as magnesium intake (mg/week) and are presented in Section 3.

### 2.11. Measurement of Hematological Ratios

To assess systemic inflammation and metabolic status, we analyzed various hematological and biochemical parameters. Neutrophil, lymphocyte, monocyte, platelet, triglyceride, and glucose levels were quantified using validated enzymatic methods. These analyses were conducted at a certified diagnostic laboratory (Alab, Poznań, Poland) following standardized procedures to ensure accuracy and reproducibility. The detailed results for these parameters have been discussed in our previous publications [28,29].

Based on the collected data, specific hematological and metabolic ratios were calculated to provide insights into inflammatory and metabolic responses. The neutrophil-to-lymphocyte ratio (NLR), monocyte-to-lymphocyte ratio (MLR), and platelet-to-lymphocyte ratio (PLR) served as indicators of immune–inflammatory system balance, with each ratio highlighting distinct aspects of systemic inflammation. Additionally, the triglyceride-to-glucose index (TyG) was calculated to evaluate insulin resistance and metabolic function [45]. The following formulas were used to compute these ratios:

- NLR = Neutrophil count (G/L)/Lymphocyte count (G/L)
- MLR = Monocyte count (G/L)/Lymphocyte count (G/L)
- PLR = Platelet count (G/L)/Lymphocyte count (G/L)
- TyG = ln [Triglyceride level (mg/dL) × Glucose level (mg/dL)/2]

### 2.12. Statistical Analysis

The Shapiro–Wilk test was employed to evaluate the normality of variable distributions. Following variance analysis (ANOVA), Tukey’s post hoc honestly significant difference test was used to identify any statistically significant differences. A significance level of 5% was set to determine statistical relevance. All statistical analyses were performed using SPSS software, version 22, for the Windows operating system. All analyses were performed on complete cases, with the per-group sample sizes specified above (sham, *n* = 8; each OVX group, *n* = 8).

## 3. Results

### 3.1. Magnesium Content, Food Intake, and Dietary Magnesium Intake

**Figure 2:**
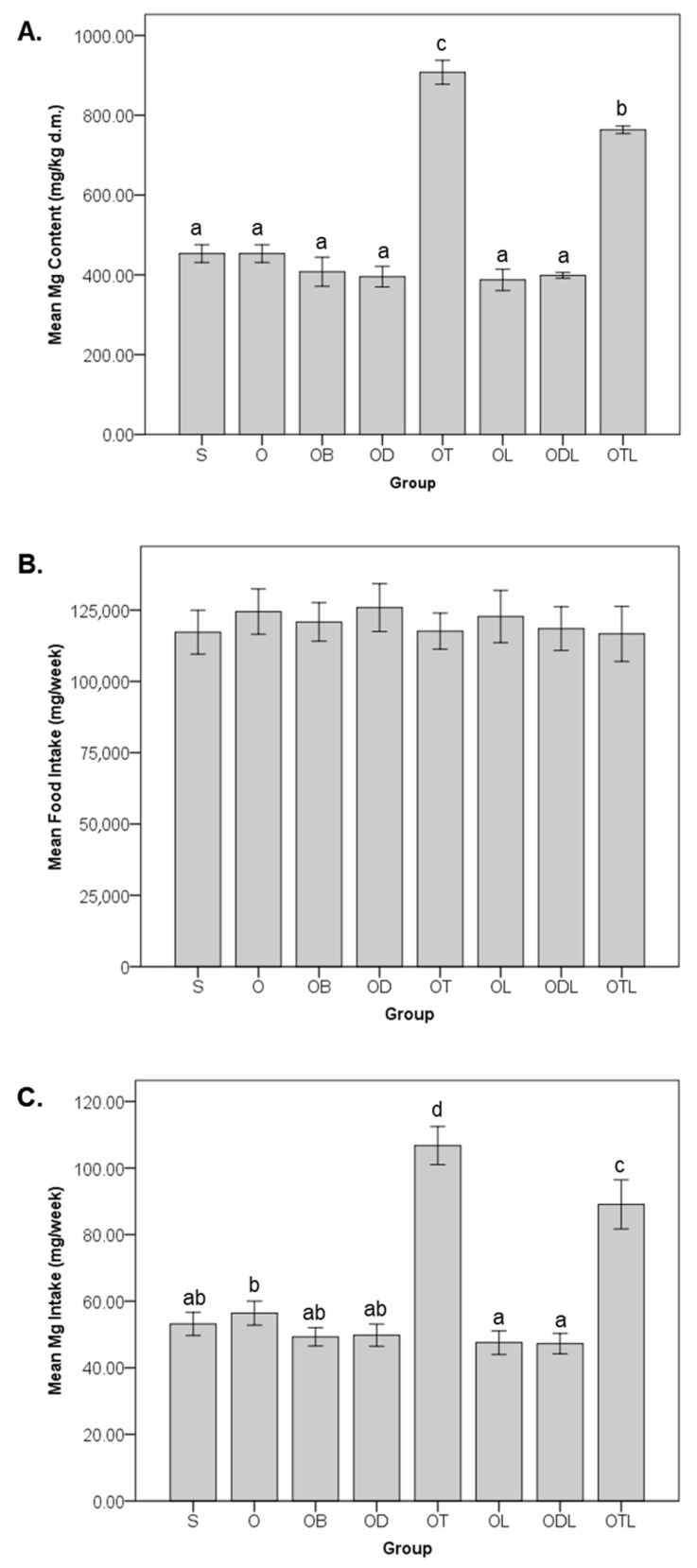
Magnesium content, food intake, and dietary magnesium intake during the six-week intervention with different modified diets. Food intake (**A**), magnesium content of the diets (**B**), and Mg intake (**C**) during the six-week intervention with different modified diets. S = sham rats fed AIN-93M; O = ovariectomized/OVX rats fed AIN-93M; OB = OVX rats fed AIN-93M with bisphosphonate; OD = OVX rats fed AIN-93M with daidzein; OT = OVX rats fed AIN-93M with tempeh; OL = OVX rats fed AIN-93M with probiotic; ODL = OVX rats fed AIN-93M with daidzein and probiotic; and OTL = OVX rats fed AIN-93M with tempeh and probiotic. The results are presented as mean ± standard deviation. Statistical analysis was performed using a one-way ANOVA, followed by Tukey’s post hoc test. Bars labelled with different letters (a, b, c, d) indicate statistically significant differences (*p* < 0.05) between the corresponding dietary groups (S, O, OB, OD, OT, OL, ODL, and OTL).

Figure 2 illustrates the magnesium content, food intake, and dietary magnesium intake across the six-week intervention with the various modified diets. The mean magnesium content in the diets (Figure 2A) exhibited significant differences among the groups. The OT group recorded the highest magnesium content (907.74 ± 29.94 mg/kg dry mass), followed by the OTL group (763.54 ± 9.36 mg/kg dry mass); these values were significantly higher than those of the other groups. No significant differences were observed in dietary magnesium content among groups S, O, OB, OD, and OL, which exhibited comparable magnesium levels. Food intake during the intervention (Figure 2B) was consistent across all experimental groups, with no significant differences being detected, indicating uniform dietary consumption among the animals irrespective of diet modification. Dietary magnesium intake (Figure 2C) was calculated as the product of magnesium concentrations in the respective diets and the weekly food consumption per rat. Intake varied significantly among the experimental groups. The OT group demonstrated the highest magnesium intake (106.76 ± 5.74 mg/week), followed by the OTL group (89.08 ± 7.38 mg/week). Both groups displayed significantly greater magnesium intake compared to all other groups. Conversely, the OL group (47.55 ± 3.55 mg/week) and ODL group (47.25 ± 3.07 mg/week) exhibited the lowest dietary magnesium intake, with no significant difference.

### 3.2. Impact on Magnesium Levels in Tissues and Feces

Table 1 summarizes the magnesium levels in the tissues and feces following a six-week intervention with different modified diets. Comparing the standard groups, magnesium levels in the spleen were more than double in the O group than in the S group. Conversely, magnesium levels in the hair samples were reduced by nearly 40% in the O group compared to the S group. No significant differences between the S and O groups were observed in the magnesium levels in the feces or liver.

Among the modified diet interventions, the OTL and OT groups showed the highest magnesium levels in the feces samples, significantly surpassing the O group. In the liver samples, magnesium levels in the OTL and OT groups were reduced by approximately 13% compared to the S group, while no significant differences were observed compared to the O group. Magnesium levels in the hair samples significantly increased in the ODL and OTL groups compared to the O group, with improvements of over 50% and 60%, respectively.

### 3.3. Impact on Hematological Ratios

**Figure 3:**
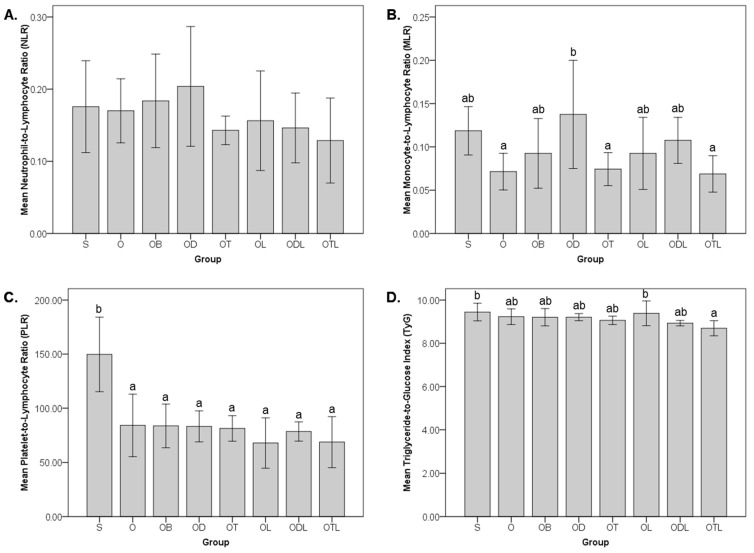
Neutrophil-to-lymphocyte ratio (NLR), monocyte-to-lymphocyte ratio (MLR), platelet-to-lymphocyte ratio (PLR), and triglyceride-to-glucose index (TyG) after the six-week intervention involving different modified diets. Neutrophil-to-lymphocyte ratio (NLR) (**A**), monocyte-to-lymphocyte ratio (MLR) (**B**), platelet-to-lymphocyte ratio (PLR) (**C**), and triglyceride-to-glucose index/TyG (**D**) after the six-week intervention administering different modified diets. S = sham rats fed AIN 93M; O = ovariectomized/OVX rats fed AIN 93M; OB = OVX rats fed AIN 93M with bisphosphonate; OD = OVX rats fed AIN 93M with daidzein; OT = OVX rats fed AIN 93M with tempeh; OL = OVX rats fed AIN 93M with probiotic; ODL = OVX rats fed AIN 93M with daidzein and probiotic; and OTL = OVX rats fed AIN 93M with tempeh and probiotic. The results are presented as mean ± standard deviation. Statistical analysis was performed using a one-way ANOVA, followed by Tukey’s post hoc test. Bars labelled with different letters (a, b) indicate statistically significant differences (*p* < 0.05) between the corresponding dietary groups (S, O, OB, OD, OT, OL, ODL, and OTL).

Figure 3 illustrates the neutrophil-to-lymphocyte ratio (NLR), monocyte-to-lymphocyte ratio (MLR), platelet-to-lymphocyte ratio (PLR), and triglyceride-to-glucose index (TyG) following a six-week intervention with modified diets. Comparing the standard groups, the O group exhibited a significant reduction in PLR, decreasing by approximately 43% compared to the S group. However, no significant differences between the S and O groups were observed in the NLR, MLR, or TyG.

NLR and PLR values showed no significant differences across interventions among the modified diet groups. In contrast, the MLR was significantly elevated in the OD group compared to the O group. For the TyG, while no significant differences were observed among the modified diet groups compared to the O group, the OTL group demonstrated a significantly lower TyG value than the S group.

### 3.4. Correlation Between Magnesium Levels and Hematological Ratios

Significant correlations between magnesium levels in fecal and tissue samples and hematological ratios are shown in Table 2. The strongest negative correlation was observed between spleen magnesium levels and the PLR (*r* = −0.626), while the strongest positive correlation was found between liver magnesium levels and TyG (*r* = 0.422). Fecal magnesium levels were significantly negatively correlated with the PLR (*r* = −0.407). Similarly, spleen magnesium levels exhibited significant negative correlations with the MLR (*r* = −0.297) and TyG (*r* = −0.289). In contrast, liver magnesium levels showed significant positive correlations with the NLR (*r* = 0.294), MLR (*r* = 0.318), PLR (*r* = 0.323), and TyG (*r* = 0.422).

## 4. Discussion

The findings of this study do not support the hypothesis that dietary interventions with tempeh, daidzein, and probiotics *L. acidophilus* significantly improve magnesium status or related hematological ratios in a postmenopausal osteoporotic rat model. While ovariectomy was associated with an increase in magnesium content in the spleen, the dietary interventions did not modify this outcome, suggesting that the changes are likely linked to estrogen deficiency rather than diet. Similarly, the reduction in PLR following ovariectomy, without further changes from different dietary groups, highlights the limited impact of these interventions on systemic inflammatory responses. The elevated MLR in the OD group points to potential pro-inflammatory effects of pure daidzein when used alone. Interestingly, tempeh’s high magnesium content promotes magnesium excretion, as seen in elevated fecal and hair levels, while paradoxically reducing magnesium levels in the liver.

Figure 2 highlights tempeh as an excellent source of magnesium, exhibiting the highest magnesium levels among all groups. The findings from this study provide valuable insights into the differential effects of dietary modifications on magnesium distribution across various tissues (Table 1). The increased magnesium levels in the feces samples from the OTL group compared to the S and O control groups suggest a higher excretion rate, potentially reflecting an improved magnesium intake and increased absorption efficiency. This finding indicates a saturation effect in which the body reaches its capacity to retain magnesium [46], leading to excess magnesium excretion with the OTL diet, which contains high magnesium levels. This observation aligns with previous evidence suggesting that dietary components, such as fermented soy and probiotics, can elevate mineral absorption efficiency [47,48,49].

The significantly lower magnesium levels in the spleen of the S group compared to all ovariectomized groups indicate that ovariectomy influences magnesium metabolism, likely through systemic changes driven by estrogen deficiency, thereby enhancing magnesium retention or redistribution to immune-related organs [50,51,52]. Despite this overall elevation in spleen magnesium content among ovariectomized groups, the modified diets, including the tempeh and probiotic combination (OTL), did not significantly alter magnesium levels compared to the O group, suggesting a saturation effect whereby magnesium uptake in immune tissues may have reached its threshold. Nonetheless, the relatively higher magnesium levels in the OTL group underscore the potential of this dietary combination to support magnesium availability in those tissues that are critical for immune regulation.

In the liver samples, it is essential to note that the magnesium levels in the OT and OTL groups were lower than those observed in the healthy group (S), indicating a potential disruption to magnesium homeostasis. This decrease in liver magnesium levels in ovariectomized rats may reflect alterations in mineral metabolism that are associated with menopausal osteoporosis, a condition in which hormonal changes, such as reduced estrogen, impact nutrient absorption, storage, and utilization [53]. In particular, the liver, as a primary organ for mineral storage [54,55], may exhibit a reduced capacity to retain magnesium in response to metabolic disturbances that are linked to osteoporosis.

Furthermore, the similar magnesium levels in hair across the ODL, OTL, and S groups suggest that dietary modifications incorporating tempeh, daidzein, and probiotics achieve a comparable effect on magnesium deposition in keratinized tissues. This finding is particularly noteworthy as previous studies have reported that higher magnesium levels in hair are significantly associated with greater BMD of the spine in premenopausal women, highlighting the potential of hair magnesium levels as a biomarker for bone health [56]. Overall, these findings point to the ability of dietary modifications to influence magnesium dynamics in ovariectomized models, reflecting both beneficial and compensatory responses to disrupted mineral metabolism that are characteristic of menopausal osteoporosis.

The unexpected finding that an ovariectomy decreased the PLR, rather than increasing the inflammation-associated ratios, warrants deeper exploration (Figure 3). This observation challenges the typical understanding of postmenopausal osteoporosis as a condition characterized by heightened systemic inflammation [57], suggesting that estrogen deficiency may elicit complex and potentially compensatory immune responses that modulate the platelet-driven inflammatory pathways [58]. However, the significant elevation in MLR observed in the OD group compared to the O group points to the pro-inflammatory potential of pure daidzein when not combined with other bioactive dietary components. While daidzein is widely recognized for its phytoestrogen content and anti-inflammatory properties [59,60], these findings underscore the limitations of using pure daidzein as a standalone supplement. Our finding aligns with evidence from a randomized trial showing that whole soy, but not purified daidzein, significantly improved LDL-C and hs-CRP levels in equol-producing postmenopausal women [61]. The enhanced efficacy of whole soy likely stems from the synergistic effects of its bioactive compounds, which are absent in isolated daidzein [61].

The TyG index, an indicator of insulin resistance and lipid metabolism [62], provides insight into the metabolic impact of dietary interventions. The observed reduction in TyG levels in the OTL group, even below the levels of the healthy sham group, suggests a modulation of lipid–glucose homeostasis by the combined effects of tempeh and probiotics. This outcome may be attributed to the actions of *L. acidophilus* in enhancing gut microbiota composition and function, which have been shown to influence insulin sensitivity and lipid metabolism through pathways such as AMPK activation and reduced oxidative stress [63]. Moreover, these effects can be attributed, in part, to the bioactive compounds produced during tempeh fermentation, which have been shown to increase levels of antioxidants, gamma-aminobutyric acid, amino acids, and phenolic acids, all of which exert significant anti-inflammatory and analgesic effects without inducing acute toxicity in animal models [64].

In contrast, the OL group exhibited the highest TyG index, indicating the potentially adverse effect of a dietary intervention consisting solely of probiotics. A similar observation was made in a previous randomized clinical study in postmenopausal women, where glucose levels were increased in the probiotic *L. acidophilus* group [65]. This outcome may be attributed to the influence of probiotics on glucose metabolism, which is possibly mediated through their interaction with the gut microbiota. Changes in gut microbiota composition can alter carbohydrate fermentation processes and the production of short-chain fatty acids, which are known to impact glucose homeostasis [66]. This finding raises important questions about the efficacy of probiotics and underscores the necessity of designing dietary strategies [67]. Moreover, it highlights the interplay between probiotics, gut microbiota, and metabolic regulation, warranting further exploration to clarify the mechanisms involved.

The correlations between magnesium levels and hematological ratios emphasize magnesium’s multifaceted role in regulating inflammation and metabolism (Table 2). The strongest negative correlation between spleen magnesium levels and the PLR (*r* = −0.626) reinforces magnesium’s anti-inflammatory properties. This relationship suggests that higher spleen magnesium levels may suppress platelet activation and lymphocyte proliferation, which are key processes linked to systemic inflammation. Magnesium’s anti-inflammatory role is further supported by its ability to modulate immune responses through its effects on cellular signaling pathways and cytokine regulation. Magnesium deficiency, for instance, has been linked to an inflammatory cascade involving endothelial cells, which are sensitive to fluctuations in extracellular magnesium concentrations [68]. Reduced magnesium levels can trigger the production of nitric oxide and vascular cell adhesion molecules, facilitating leukocyte adhesion and recruitment during inflammation. Additionally, low magnesium has been associated with elevated IL-1α and IL-6, key cytokines involved in acute inflammatory responses [68]. These findings agree that sufficient magnesium levels are essential for maintaining vascular integrity and modulating immune responses, particularly under inflammatory conditions.

Interestingly, spleen magnesium levels demonstrated significant negative correlations with the MLR (*r* = −0.297) and TyG (*r* = −0.289), suggesting that magnesium may play an essential role in mitigating inflammation and supporting metabolic regulation. Elevated magnesium levels in the spleen appear to be inversely associated with certain markers of metabolic disorders, suggesting that magnesium’s anti-inflammatory and insulin-sensitizing properties extend to immune-related tissues. These correlations align with magnesium’s established functions in reducing oxidative stress and stabilizing immune cell membranes. Magnesium deficiency exacerbates oxidative stress by promoting the accumulation of reactive oxygen species (ROS), primarily through mitochondrial dysfunction, disrupted calcium homeostasis, and activation of the renin–angiotensin–aldosterone system. This increase in ROS, coupled with elevated pro-inflammatory cytokines like IL-1, IL-6, and TNF-α, creates a cycle of inflammation and oxidative stress that can heighten the risk of chronic diseases [69]. Furthermore, magnesium deficiency also impairs insulin signaling, which may contribute to insulin resistance by disrupting certain key pathways, such as the insulin receptor substrate-1, its interaction with phosphatidyl-inositol 3 kinase, and reduced Akt phosphorylation [69]. These findings suggest that maintaining adequate magnesium levels is crucial for regulating both immune responses and metabolic processes, potentially helping to prevent the escalation of inflammation and metabolic dysfunction.

Conversely, the observed positive correlation between liver magnesium levels and TyG (*r* = 0.422) underscores magnesium’s critical role in liver-driven metabolic processes, particularly those regulating lipid and glucose metabolism. This relationship suggests that while magnesium is critical for metabolic homeostasis, its elevated levels in the liver may reflect compensatory mechanisms that are linked to metabolic dysregulation, such as insulin resistance [70]. Interestingly, the decreased liver magnesium levels in ovariectomized rats raise questions about the interplay between magnesium status, estrogen deficiency, and metabolic disorders. Reduced liver magnesium levels in this context could signify impaired magnesium distribution, potentially exacerbating the metabolic disturbances common in postmenopausal conditions. The liver is a central organ in maintaining systemic energy homeostasis, and higher magnesium levels in this organ likely reflect improved metabolic function [71]. This relationship also suggests that the liver may serve as a reservoir for magnesium redistribution during periods of metabolic stress or dysregulation. Furthermore, the positive correlations of liver magnesium levels with the NLR (*r* = 0.294), MLR (*r* = 0.318), and PLR (*r* = 0.323) highlight a complex dynamic wherein liver magnesium levels are indicative not only of metabolic demands but also of the body’s inflammatory status. This interplay is particularly relevant in energy metabolism, where magnesium modulates key pathways such as those regulated by AMPK, PGC-1α, and PPARα, which govern lipid oxidation and mitochondrial function in the liver. Disruptions in these pathways can lead to hepatic steatosis and NAFLD, with inflammation playing a pivotal role. Activation of inflammatory mediators such as NF-κB and TLR4 signaling in the liver further emphasizes the connection between metabolic dysregulation and inflammation, reinforcing the need for effective magnesium management to preserve both metabolic and immune homeostasis [71].

The inclusion of alendronate bisphosphonate (group OB) at a dose of 3 mg/kg body weight weekly in the dietary intervention did not exhibit any significant impact on magnesium levels across tissues, feces, or hematological ratios (Table 1, Figure 3). This result suggests that while alendronate is well-recognized for its efficacy in preventing bone resorption and treating osteoporosis [72,73], its effects on magnesium metabolism and systemic inflammation may be negligible in this study. The OB group demonstrated outcomes comparable to the ovariectomized control group (O), which was fed a standard diet. This finding indicates that alendronate alone may not influence magnesium bioavailability or its physiological distribution in this context.

This study encompasses several strengths, including integrating a comprehensive dietary intervention involving tempeh, daidzein, probiotics, and their combinations, which were systematically compared with alendronate bisphosphonate treatment. This approach allowed for a robust evaluation of their effects on magnesium metabolism, hematological indices, and systemic inflammation. Using an ovariectomized rat model provided a well-established and controlled platform by which to simulate postmenopausal osteoporosis and investigate the efficacy of dietary interventions. Furthermore, the meticulous design ensured uniform food intake across all groups, minimizing confounding variables and enhancing the reliability of the observed effects. The study also addressed key gaps in the literature, such as the interplay between magnesium levels and hematological ratios, advancing our understanding of dietary bioactive elements and how they modulate metabolic and inflammatory responses.

While our findings reveal promising trends in magnesium bioavailability and its impact on hematological and metabolic parameters, certain limitations warrant consideration. Regarding the constraints, the study would have benefited from including additional biomarkers of inflammatory and metabolic alterations, such as cytokines, oxidative stress markers, and lipid profiles, to provide a more comprehensive understanding of the systemic effects of dietary interventions. Inflammatory biomarkers, for instance, can reveal critical insights into the low-grade inflammation linked to metabolic syndrome and osteoporosis [74,75]. Additionally, exploring magnesium transporters and their expression in key tissues such as the liver and spleen would have strengthened our mechanistic understanding of magnesium regulation. Magnesium transporters play a pivotal role in cellular magnesium homeostasis and could provide a deeper understanding of the observed tissue-specific effects [76]. Furthermore, different dose levels of daidzein, tempeh, or probiotics were not evaluated in this study, which limits the ability to determine the most effective or the minimal effective intake. Although a six-week intervention is sufficient to capture early biological responses, longer feeding periods would be required to assess sustained or chronic effects on bone density and metabolic outcomes. Future studies should, therefore, consider extended interventions and include dose–response comparisons to better define optimal intake levels and the long-term impacts of these dietary components.

Another limitation is the lack of data on gut microbiota composition and its metabolites, which are known to influence both magnesium absorption and systemic inflammation. Gut microbiota-mediated metabolites, such as short-chain fatty acids, are increasingly recognized as key players in the modulation of mineral bioavailability and immune responses [77]. Future studies should integrate these aspects to provide a holistic view of the interaction between dietary bioactive compounds, magnesium metabolism, and systemic health.

Additionally, there is a critical need to explore the long-term effects of dietary magnesium interventions on hematological and metabolic markers in both preclinical and clinical settings. Longitudinal studies are essential to assess whether the observed improvements in inflammatory indices, glucose regulation, and magnesium bioavailability are sustained over time and how they influence disease prevention or progression. Such studies should also evaluate the cumulative effects of magnesium-rich diets on multi-organ health and mineral homeostasis.

Clinical studies are particularly warranted to validate these preclinical findings and establish their translatability to human populations. Well-designed randomized controlled trials involving populations at risk of magnesium deficiency or those with metabolic disorders, such as postmenopausal women or individuals with metabolic syndrome, would be invaluable. These trials should integrate advanced tools, such as gut microbiome profiling, metabolomics, and magnesium transport analysis, to capture the complex interactions between magnesium, dietary bioactive compounds, and systemic health. Future research can pave the way for evidence-based dietary recommendations and therapeutic strategies for magnesium-related health benefits by addressing these knowledge gaps.

## 5. Conclusions

The authors of this study conclude that while ovariectomy significantly altered magnesium status and hematological ratios, the dietary combination of tempeh, daidzein, and probiotics did not demonstrate a clear beneficial effect on magnesium status or inflammatory ratios in a postmenopausal osteoporotic rat model. While alendronate bisphosphonate showed limited impact on magnesium levels and hematological ratios, the dietary combinations explored in this study offer a promising alternative for addressing magnesium imbalance and associated systemic effects. The correlations between magnesium levels and hematological ratios further highlight their role in promoting bone health and immune function. These findings pave the way for further investigations into the mechanisms driving these effects and their potential applicability in human clinical settings.

## Figures and Tables

**Table 1:** Magnesium levels in collected tissues and feces after the six-week intervention involving administering different modified diets.

Group	Tissues						
	Faeces (mg/g)	Spleen (μg/g)	Femur (mg/g)	Heart (μg/g)	Liver (μg/g)	Kidney (μg/g)	Hair (μg/g)
S	12.41 ± 1.53 ^ab^	360.19 ± 113.13 ^a^	5.61 ± 0.74	774.17 ± 20.05	658.84 ± 52.26 ^b^	627.80 ± 125.12	81.33 ± 28.16 ^b^
O	10.51 ± 2.18 ^a^	747.66 ± 106.82 ^b^	5.05 ± 1.24	712.89 ± 58.96	647.00 ± 59.36 ^ab^	625.61 ± 90.68	50.69 ± 5.41 ^a^
OB	12.42 ± 1.58 ^ab^	689.54 ± 125.27 ^b^	5.95 ± 0.14	686.80 ± 35.28	619.21 ± 59.78 ^ab^	630.24 ± 87.14	47.61 ± 9.77 ^a^
OD	14.40 ± 2.82 ^abc^	683.80 ± 39.96 ^b^	5.93 ± 0.24	736.17 ± 115.00	613.99 ± 16.64 ^ab^	593.00 ± 39.84	43.78 ± 4.60 ^a^
OT	17.36 ± 1.46 ^bc^	691.17 ± 42.83 ^b^	5.88 ± 0.30	682.72 ± 21.42	570.57 ± 42.19 ^a^	599.04 ± 32.16	46.24 ± 10.06 ^a^
OL	11.83 ± 3.13 ^a^	683.93 ± 105.86 ^b^	5.77 ± 0.41	699.89 ± 69.74	649.40 ± 84.22 ^ab^	589.13 ± 26.37	65.14 ± 18.63 ^ab^
ODL	11.85 ± 2.24 ^a^	652.11 ± 41.96 ^b^	5.37 ± 0.19	720.30 ± 24.65	584.39 ± 24.67 ^ab^	704.70 ± 26.65	76.94 ± 12.22 ^b^
OTL	19.32 ± 2.26 ^c^	752.28 ± 60.14 ^b^	5.45 ± 0.25	721.36 ± 45.72	570.36 ± 30.79 ^a^	624.90 ± 40.58	82.08 ± 10.92 ^b^

S = sham rats fed AIN 93M; O = ovariectomized/OVX rats fed AIN 93M; OB = OVX rats fed AIN 93M with bisphosphonate; OD = OVX rats fed AIN 93M with daidzein; OT = OVX rats fed AIN 93M with tempeh; OL = OVX rats fed AIN 93M with probiotic; ODL = OVX rats fed AIN 93M with daidzein and probiotic; and OTL = OVX rats fed AIN 93M with tempeh and probiotic. The results were analyzed using an ANOVA followed by Tukey’s post hoc test for statistically significant differences, highlighting significant variations among the dietary groups. Data are shown as mean ± standard deviation. Values labelled with different letters (a, b, c) indicate statistically significant differences (*p* < 0.05).

**Table 2:** Pearson’s correlation of magnesium levels and hematological ratios.

Correlation	Correlation Coefficients (*r*)	Significant Values (*p*)
Mg Spleen—MLR	−0.297	0.025 *
Mg Spleen—TyG	−0.289	0.034 *
Mg Spleen—PLR	−0.626	0.000 **
Mg Liver—NLR	0.294	0.024 *
Mg Liver—MLR	0.318	0.015 *
Mg Liver—PLR	0.323	0.014 *
Mg Liver—TyG	0.422	0.001 **

Mg = Magnesium; NLR = neutrophil-to-lymphocyte ratio; MLR = monocyte-to-lymphocyte ratio; PLR = platelet-to-lymphocyte ratio; and TyG = triglyceride-to-glucose index. Significant *p*-values are marked with an asterisk (*) for values less than 0.05 and with two asterisks (**) for values below 0.01. For clarity, only statistically significant correlations are included, while non-significant correlations have been excluded.

## Data Availability

All data generated or analyzed during this study are available from the corresponding author on reasonable request.

## Abbreviations

The following abbreviations are used in this manuscript:

AMPK	Adenosine monophosphate-activated protein kinase
BMD	Bone mineral density
IL-1	Interleukin-1
IL-6	Interleukin-6
hsCRP	High-sensitivity C-reactive protein
LDL	Low-density lipoprotein
MLR	Monocyte-to-lymphocyte ratio
NAFLD	Non-alcoholic fatty liver disease
NF-κB	Nuclear factor kappa B
NLR	Neutrophil-to-lymphocyte ratio
PGC-1α	Peroxisome proliferator-activated receptor-gamma coactivator (PGC)-1alpha
PLR	Platelet-to-lymphocyte ratio
PPARα	Peroxisome proliferator-activated receptor-alpha
ROS	Reactive oxygen species
TLR4	Toll-like receptor 4
TNF-α	Tumor necrosis factor-alpha
TyG	Triglyceride-to-glucose index
